# Erector spinae muscle-based nomogram for predicting in-hospital mortality among older patients with severe community-acquired pneumonia

**DOI:** 10.1186/s12890-023-02640-z

**Published:** 2023-09-14

**Authors:** Na Shang, Qiujing Li, Huizhen Liu, Junyu Li, Shubin Guo

**Affiliations:** 1 Department of Emergency Medicine, Beijing Chao-Yang Hospital, Capital Medical University, Beijing Key Laboratory of Cardiopulmonary Cerebral Resuscitation, Beijing, 100020 China; 2 Department of Emergency Medicine, Capital Medical University School of Rehabilitation Medicine, Beijing Bo’Ai Hospital, China Rehabilitation Research Center, Beijing, 100068 China; 3 Department of Emergency Medicine, Capital Medical University, Beijing Shijitan Hospital, Beijing, 100038 China

**Keywords:** Severe community-acquired pneumonia, Aged, nomogram, Erector spinae muscle, Mortality

## Abstract

**Background:**

No multivariable model incorporating erector spinae muscle (ESM) has been developed to predict clinical outcomes in older patients with severe community-acquired pneumonia (SCAP). This study aimed to construct a nomogram based on ESM to predict in-hospital mortality in patients with SCAP.

**Methods:**

Patients aged ≥ 65 years with SCAP were enrolled in this prospective observational study. Least absolute selection and shrinkage operator and multivariable logistic regression analyses were used to identify risk factors for in-hospital mortality. A nomogram prediction model was constructed. The predictive performance was evaluated using the concordance index (C-index), calibration curve, net reclassification improvement (NRI), integrated discrimination improvement (IDI), and decision curve analysis.

**Results:**

A total of 490 patients were included, and the in-hospital mortality rate was 36.1%. The nomogram included the following independent risk factors: mean arterial pressure, peripheral capillary oxygen saturation, Glasgow Coma Scale score (GCS), lactate, lactate dehydrogenase, blood urea nitrogen levels, and ESM cross-sectional area. Incorporating ESM into the base model with other risk factors significantly improved the C-index from 0.803 (95% confidence interval [CI], 0.761–0.845) to 0.836 (95% CI, 0.798–0.873), and these improvements were confirmed by category-free NRI and IDI. The ESM-based nomogram demonstrated a high level of discrimination, good calibration, and overall net benefits for predicting in-hospital mortality compared with the combination of confusion, urea, respiratory rate, blood pressure, and age ≥ 65 years (CURB-65), Pneumonia Severity Index (PSI), Acute Physiology and Chronic Health Evaluation II (APACHEII), and Sequential Organ Failure Assessment (SOFA).

**Conclusions:**

The proposed ESM-based nomogram for predicting in-hospital mortality among older patients with SCAP may help physicians to promptly identify patients prone to adverse outcomes.

**Trial registration:**

This study was registered at www.chictr.org.cn (registration number Chi CTR-2300070377).

**Supplementary Information:**

The online version contains supplementary material available at 10.1186/s12890-023-02640-z.

## Background

With increasing life expectancy and decreasing birth rates, the proportion of adults aged 65 years and over will rise dramatically to 38% by the year 2050 [1]. Community-acquired pneumonia (CAP) is the leading cause of death among older patients, and its incidence and mortality gradually increase with age [2–4]. Severe CAP (SCAP) is a major challenge in intensive care units (ICU); hospital mortality rates range from 25% to more than 50% and are higher among older adults [5, 6]. Mortality related to SCAP remains staggeringly high despite advances in rapid diagnosis, antibiotic coverage, and vaccine inoculation [7]. The risk factors for mortality associated with SCAP include patient-related factors (e.g., age, comorbidities, vital signs), pathogen specificity, severity of illness, and related management processes (e.g., delays in ICU care) [5]. Identification of risk factors at an early stage is crucial to prevent unnecessary mortality and enable timely intervention.

Sarcopenia is characterized by an age-related decline in skeletal muscle, muscle strength, and/or physical performance [8], which may contribute to a host of negative outcomes, including reduced functional capacity, falls, and even death in older people. A variety of imaging techniques are available for assessing muscle mass including dual-energy X-ray absorptiometry, bioelectrical impedance analysis, magnetic resonance imaging, and computed tomography (CT) [9]. The cross-sectional area of the skeletal muscles at the level of the third lumbar vertebra on abdominal CT is conventionally used to investigate skeletal muscle characteristics [10]. However, abdominal CT is not routinely performed in patients with respiratory diseases. Many studies have confirmed that the evaluation of thoracic skeletal muscles via chest CT can be used to measure sarcopenia. Recent studies have demonstrated that chest CT-derived cross-sectional areas of erector spinae muscles (ESMcsa) at the 12th thoracic level are associated with poor prognosis in patients with chronic obstructive pulmonary disease, severe novel coronavirus disease 2019 (COVID-19), and other respiratory diseases [11, 12]. Furthermore, ESMcsa may serve as a prognostic indicator for in-hospital mortality among patients with CAP or SCAP [13–15].

To date, there is no reliable evaluation tool for predicting adverse outcomes of SCAP in a timely manner. The predictive effects of laboratory biomarkers such as C-reactive protein and procalcitonin have been suggested as inadequate for clinical use in older patients with SCAP [16]. Pneumonia severity scores such as the combination of confusion, urea, respiratory rate, blood pressure, and age ≥ 65 years (CURB-65); Pneumonia Severity Index (PSI); and critical illness scores such as the Acute Physiology and Chronic Health Evaluation II (APACHE II) and Sequential Organ Failure Assessment (SOFA), among others, may not be accurate or applicable in emergency departments due to the complexity and time-consuming nature of the tests [17].

A chest CT, performed for diagnosis or assessment of therapeutic effects, is a routine examination modality for patients with SCAP, and ESMcsa is easily determined using chest CT without extra cost or radiation. However, to the best of our knowledge, no multivariable model incorporating ESMcsa has been developed to predict clinical outcomes in older patients with SCAP. Therefore, in the current study, we aimed to use basic clinical and laboratory indicators on admission, as well as ESMcsa determined from chest CT, to construct a simple ESM-based nomogram for predicting in-hospital mortality among older patients with SCAP. We then compared the predictive value of the novel nomogram with widely used scoring systems.

## Methods

### Study design and patients

This prospective observational cohort study included older patients (aged ≥ 65 years) with SCAP who were admitted to Beijing Chao-Yang Hospital between January 1, 2022, and November 30, 2022. During the study period, patients those who tested negative for COVID-19 were admitted. This study was approved by the Institutional Review Board of Beijing Chao-Yang Hospital Capital Medical University (2022-ke-430) and conducted in accordance with the Declaration of Helsinki and its later amendments. Written informed consent was obtained from each patient or their next of kin. We followed the TRIPOD (transparent reporting of a multivariable prediction model for individual prognosis or diagnosis) guidelines [18] for our prognostic model and a completed TRIPOD checklist was provided as an additional file (Additional file 1).

The method of events per variable (EPV) was used to estimate the sample size. An EPV of 10 meant that each independent variable required 10 positive events. In this study, at least 100 positive events were needed if 10 candidate variables were expected to be included in the multivariable logistic regression model. Since the mortality rate of older patients with SCAP was reported approximately 30% [7], the sample size was at least 333. Considering the situation of missing data, an increase of 20% was required and the overall sample size was 400. A total of 490 older SCAP patients were enrolled in the current study.

The inclusion criteria were as follows: (1) age ≥ 65 years, and (2) a clinical diagnosis of SCAP according to the Infectious Diseases Society of America/American Thoracic Society consensus guidelines [19]. SCAP was defined as fulfilment of at least one major criterion (septic shock with need for vasopressors, requirement for mechanical ventilation) or three minor criteria (respiratory rate ≥ 30 breaths/min, partial pressure of oxygen/fraction of inspired oxygen ratio ≤ 250, multilobar infiltrates, confusion/disorientation, blood urea nitrogen [BUN] level ≥ 20 mg/dL, white blood cell count < 4000 cells/µL, platelet count < 100,000/µL, hypothermia-core temperature < 36℃, hypotension requiring aggressive fluid resuscitation).

The exclusion criteria were as follows: (1) no chest CT scan within 24 h after hospitalization, (2) pre-existing or acute peripheral neuromuscular diseases, (3) history of thoracic vertebral surgery, (4) imaging data not of high quality, (5) incomplete clinical data, (6) repeated admission, (7) severe immunosuppression or immunosuppressive therapy, and (8) discharge within 24 h. All patients received standard therapeutic strategies based on the guidelines for SCAP during hospitalization [20].

### Data collection

Demographic variables, including age, sex, and body mass index (BMI); vital signs on admission (temperature, heart rate, respiratory rate, mean arterial pressure [MAP], and peripheral capillary oxygen saturation [SpO_2_]); and chronic diseases (hypertension, diabetes mellitus, coronary artery disease, chronic kidney disease, cerebrovascular disease, chronic liver disease, chronic pulmonary disease, and cancer) were collected. Baseline ESMcsa, complete blood counts, biochemical parameters, coagulation indicators, and arterial blood gas analysis results were measured within 24 h of admission. Mechanical ventilation and vasoactive agents were not evaluated in our study because these interventions may not have been performed on admission. The CURB-65, PSI, APACHE II, and SOFA scores were independently assessed by two emergency physicians. Functional and frailty statuses were evaluated using the Barthel index and Clinical Frailty Scale (CFS), respectively. All data were recorded within 24 h of admission.

### Chest CT scans

Plain chest CT scans were performed and reconstructed using a 1.25-mm slice thickness. The presence of hydrothorax and multilobar infiltration was noted, and ESMcsa was determined based on the chest CT. The quantitative assessment of ESMcsa was conducted using an AW Volume Share 7 workstation (GE Medical Systems S.C.S) retrieved from the institutional Picture Archiving and Communication System. The CT histogram software X Section was used to manually delineate the region of interest (ROI); this software calculates the ROI area automatically. The Hounsfield unit (HU) thresholds were set from –29 HU to + 150 HU, which are consistent with skeletal muscle density. ESMcsa was measured at the inferior margin of the 12th thoracic vertebra within 24 h. The bilateral ESMcsa values were summed to determine the total ESMcsa value. All measurements were obtained, and the averages were calculated by one of two trained emergency physicians.

### Study outcomes

The primary outcome was all-cause mortality during hospitalization.

### Statistical analysis

Continuous variables were presented as medians (interquartile ranges [IQR]) for non-normally distributed variables and as means (standard deviations) for normally distributed variables. Categorical variables were presented as numbers (percentages). Baseline characteristics were compared between survivors and non-survivors using the Mann–Whitney U test, Student’s t-test, or chi-square test, as appropriate. Multiple imputations were used to account for missing variables if the missing values were < 20%. Least absolute selection and shrinkage operator (LASSO) and multivariable logistic regressions were applied to select possible predictive variables, and odds ratios (ORs) with 95% confidence intervals (CIs) were calculated. A forward stepwise selection procedure was performed; the independent variables remaining in the multivariable logistic regression model were used to construct the nomogram. The discriminatory ability of the nomogram was assessed using the concordance index (C-index) (also named the area under the receiver operating characteristic curve [AUC]), and 1000 bootstrap resamples were used for internal validation. A calibration plot was constructed, and the Hosmer–Lemeshow goodness-of-fit test and Brier score were calculated. Decision curve analysis (DCA) was used to measure the net benefit of the nomogram. The C-index and DCA of the nomogram were compared with those of the base model, CURB-65, PSI, APACHE II, and SOFA. Net reclassification improvement (NRI) and integrated discrimination improvement (IDI) were used to compare the discrimination slopes. All data were analyzed using SPSS (version 26.0; SPSS Inc., Chicago, IL, USA) and R software (version 4.2.0; The R Foundation for Statistical Computing). All *P*-values were two-tailed, and *P* < 0.05 was considered statistically significant.

## Results

### Baseline characteristics

**Fig. 1:**
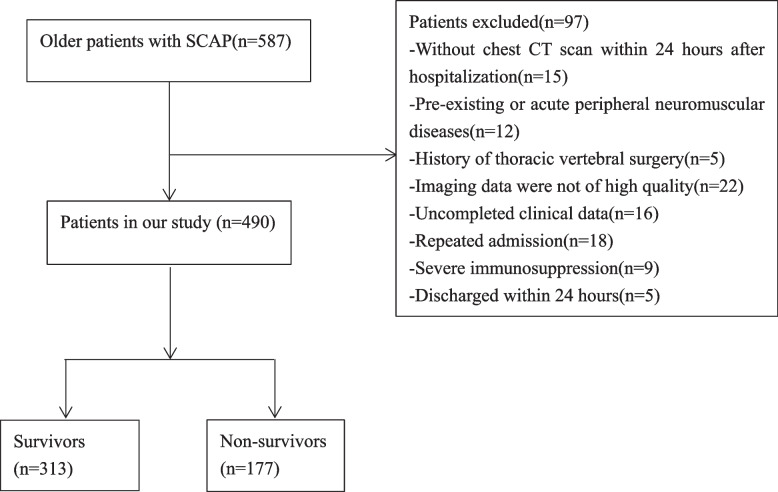
Flow chart of the enrollment of patients in this study

A total of 490 older patients with SCAP were included in this study based on the inclusion and exclusion criteria (Fig. 1). The overall in-hospital mortality rate was 36.1% (177/490). Among all patients, 293 (59.8%) were men, the median age was 80.0 years (IQR, 16.0), and the median BMI was 23.0 kg/m^2^ (IQR, 3.7). The baseline data are shown in Table 1. There were significant differences in age, heart rate, MAP, SpO_2_, ESMcsa, comorbidities (including diabetes mellitus, cerebrovascular disease, chronic pulmonary disease, and cancer), and the Glasgow Coma Scale (GCS), Barthel index, CFS, CURB-65, PSI, APACHE II, and SOFA scores between survivors and non-survivors.

**Table 1:** Baseline characteristics of participants

Parameter	Overall (*n* = 490)	Survivors (*n* = 313)	Non-survivors (*n* = 177)	*P*
Age, year, median(IQR)	80(16)	78(17)	82(15)	0.012
Male, n(%)	293(59.8)	183(62.5)	110(37.5)	0.425
BMI, kg/m^2^, mean (SD)	23.0(3.7)	23.0(3.8)	22.4(4.1)	0.093
Temperature, ℃, median(IQR)	36.5(1.7)	36.5(1.7)	36.5(1.7)	0.991
Heart rate, times/min, median(IQR)	97(30)	95(31)	102(32)	0.002
Respiratory rate, times/min, median(IQR)	18(4)	18(3)	18(4)	0.053
MAP, mmHg, median(IQR)	91.3(23.8)	93.7(24.3)	86.7(26.3)	< 0.001
SpO2, %, median(IQR)	95(10)	95(9)	92(13)	0.016
ESMcsa, cm^2^, mean(SD)	26.0(11.3)	27.8(11.1)	22.1(9.22)	< 0.001
Hypertension (n,%)	293(59.8)	193(65.9)	100(34.1)	0.263
Diabetes mellitus (n,%)	180(36.7)	126(70)	54(30)	0.032
Coronary artery disease (n,%)	166(33.9)	105(63.3)	61(36.7)	0.837
Chronic kidney disease	93(19.0)	60(64.5)	33(35.5)	0.887
Cerebrovascular disease	144(29.4)	81(56.3)	63(43.7)	0.023
Chronic liver disease	21(4.3)	16(76.2)	5(23.8)	0.230
Chronic pulmonary disease	89(18.2)	65(73)	24(27)	0.047
Cancer	84(17.1)	43(51.2)	41(48.8)	0.008
GCS, median(IQR)	15(7)	15(4)	9(10)	< 0.001
Barthel index, median(IQR)	53(75)	60(70)	30(70)	< 0.001
CFS, median(IQR)	6(3)	6(3)	7(2)	< 0.001
CURB-65, median(IQR)	3(1)	2(1)	3(2)	< 0.001
PSI, mean(SD)	137(32)	128(27)	153(35)	< 0.001
APACHEII, median(IQR)	16(8)	14(7)	20(10)	< 0.001
SOFA, median(IQR)	5(4)	4(3)	7(4)	< 0.001

*Abbreviations: SD* Standard deviation, *BMI* Body mass index, *IQR* Interquartile range, *MAP* Mean arterial pressure, *SpO2* Peripheral capillary oxygen saturation, *ESMcsa* Cross-sectional area of erector spinae muscle, *GCS* Glasgow coma scale, *ADL* Ability of daily living, *CFS* Clinical frailty scale, *PSI* Pneumonia Severity Index, *APACHEII* Acute Physiology and Chronic Health Evaluation II, *SOFA* Sequential Organ Failure Assessment

### Development of the nomogram

**Fig. 2:**
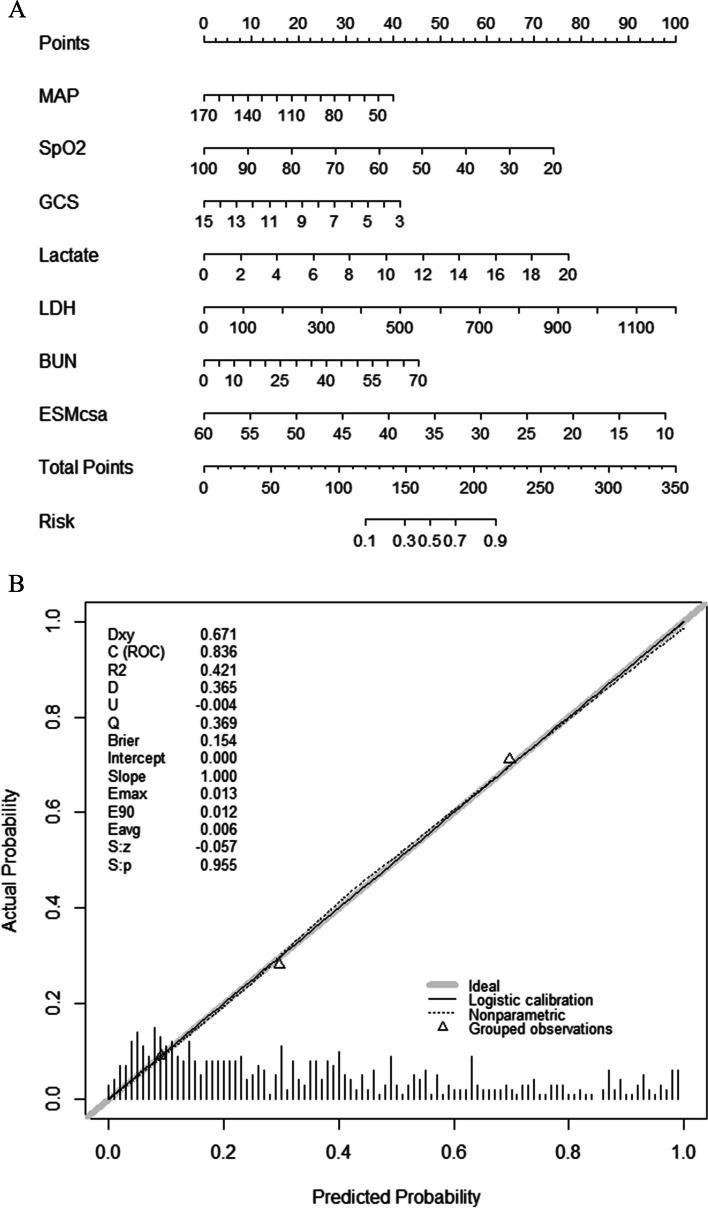
**A** The nomogram for in-hospital mortality in older patients with SCAP. Instructions of the nomogram: The points corresponding to each variable were displayed on the top of the nomogram and the sum of the points of these seven variables was calculated as the total points. At the bottom of the nomogram, the risk corresponding to the total points was the probability of in-hospital mortality in older patients with SCAP. MAP, mean arterial pressure; SpO2, peripheral capillary oxygen saturation; GCS, glasgow coma scale; LDH, lactate dehydrogenase; BUN, blood urea nitrogen; ESMcsa, cross-sectional area of erector spinae muscle. **B** Calibration curve of nomogram

The LASSO regression model included 59 candidate variables measured upon admission: age, sex, BMI, vital signs, comprehensive geriatric assessment, comorbidities, arterial blood gas analysis results, complete blood cell count, liver and renal function, electrolyte concentrations, coagulation test results, procalcitonin concentration, hydrothorax, multilobar infiltration, and ESMcsa. Further LASSO regression analysis resulted in the identification of 10 variables that were associated with in-hospital mortality: MAP, SpO_2_, GCS, CFS, red blood cell distribution width, ESMcsa, and lactate dehydrogenase (LDH), lactate, BUN, and potassium levels. The 10 variables were entered into multivariable logistic regression models, and seven variables remained statistically significant. A nomogram model was developed using these seven predictors: MAP (OR, 0.986; 95% CI, 0.974–0.998; *P* = 0.023), SpO_2_ (OR, 0.959; 95% CI, 0.937–0.982; *P* < 0.001), GCS (OR, 0.855; 95% CI, 0.809–0.903; *P* < 0.001), lactate (OR, 1.191; 95% CI, 1.047–1.354; *P* = 0.008), LDH (OR, 1.004; 95% CI, 1.002–1.005; *P* < 0.001), BUN (OR, 1.030; 95% CI, 1.008–1.052; *P* = 0.007), and ESMcsa (OR, 0.915; 95% CI, 0.886–0.946; *P* < 0.001) (Table 2). According to the nomogram, each predictor corresponded to a point and the total points was obtained by summing them. The predictive risk corresponding to the total points was the probability of in-hospital mortality in older patients with SCAP (Fig. 2A).

**Table 2:** Risk factors associated with in-hospital mortality

Risk factors	Univariable analysis		Multivariable analysis	
	OR (95% CI*)*	*P* value	OR (95% CI)	*P* value
MAP	0.982(0.972–0.992)	< 0.001	0.986(0.974–0.998)	0.023
SpO2	0.970(0.952–0.988)	0.001	0.959(0.937–0.982)	< 0.001
GCS	0.841(0.803–0.880)	< 0.001	0.855(0.809–0.903)	< 0.001
CFS	1.586(1.384–1.818)	< 0.001	-	
Lactate	1.339(1.201–1.493)	< 0.001	1.191(1.047,1.354)	0.008
RDW	1.209(1.121–1.304)	< 0.001	-	
LDH	1.004(1.002–1.005)	< 0.001	1.004(1.002–1.005)	< 0.001
BUN	1.038(1.020–1.056)	< 0.001	1.030(1.008–1.052)	0.007
Potassium	1.343(1.100–1.640)	0.004	-	
ESMcsa	0.903(0.878–0.929)	< 0.001	0.915(0.886–0.946)	< 0.001

*Abbreviations: MAP* Mean arterial pressure, *SpO2* Peripheral capillary oxygen saturation, *GCS* Glasgow coma scale, *CFS* Clinical frailty scale, *RDW* Red blood cell distribution width, *LDH* Lactate dehydrogenase, *BUN* Blood urea nitrogen, *ESMcsa* Cross-sectional area of erector spinae muscle

### Performance of the nomogram

**Fig. 3:**
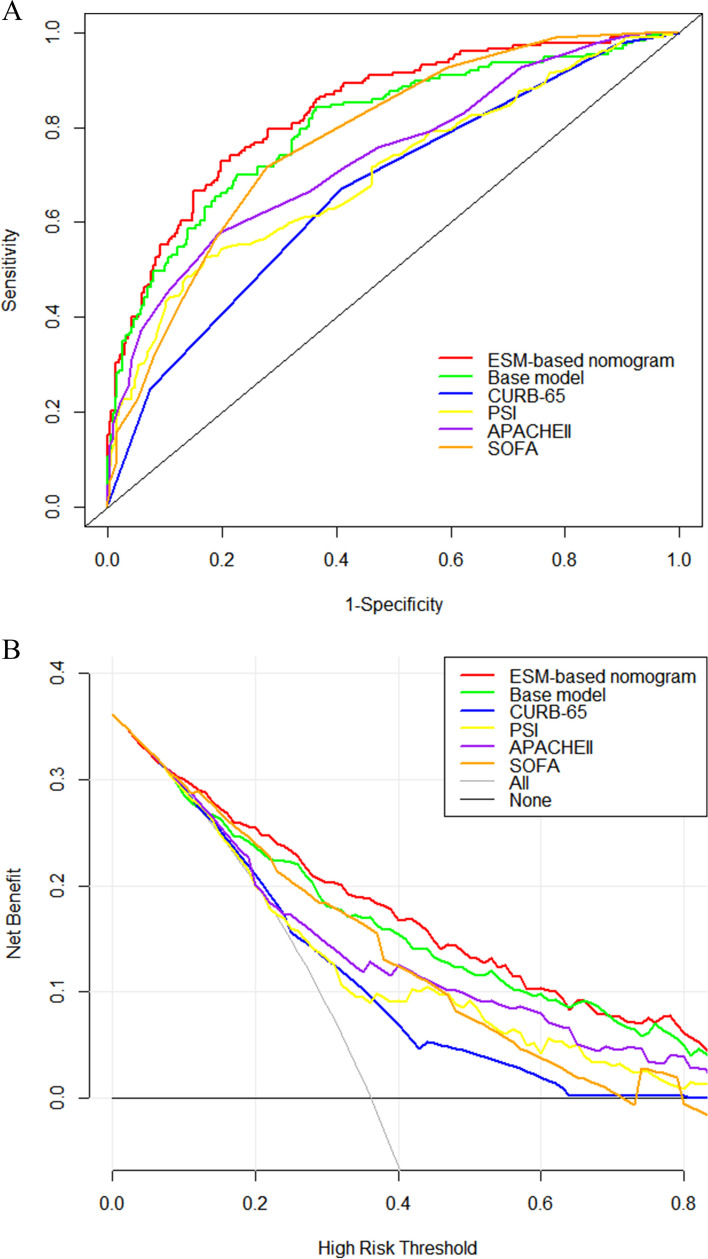
**A** The ROC curves of ESM-based nomogram, Base model, CURB-65, PSI, APACHEII and SOFA. **B** The DCA curves of ESM-based nomogram, Base model, CURB-65, PSI, APACHEII and SOFA

The C-index of the base model (with risk factors including MAP, SpO_2_, GCS, LDH, lactate, and BUN) was 0.803 (95% CI, 0.761–0.845). Compared with that of the base model, the ESM-based nomogram significantly improved the prediction of in-hospital mortality and had a C-index of 0.836 (95% CI, 0.798–0.873; *P* = 0.005), an NRI of 0.132 (95% CI, 0.044–0.217; *P* = 0.003), and an IDI of 0.051 (95% CI, 0.030–0.071; *P* < 0.001). The C-index indicated that the nomogram had good prediction accuracy for in-hospital mortality. The bias-corrected C-index was 0.826 based on bootstrapping validation. The calibration curve indicated good consistency between the predicted and observed probabilities with a Brier score of 0.154 (Fig. 2B). The Hosmer–Lemeshow goodness-of-fit test showed a chi-square of 0.700 and a *P-*value of 0.705. The predictive value of the ESM-based nomogram was higher than that of the CURB-65, PSI, APACHE II, and SOFA scoring systems, which had C-indices of 0.669 (95% CI, 0.622–0.715), 0.704 (95% CI, 0.654–0.754), 0.740 (95% CI, 0.693–0.786), and 0.778 (95% CI, 0.737–0.819), respectively. In addition, NRI and IDI showed that the novel ESM-based nomogram exhibited a superior performance compared with that of the other scoring systems (Fig. 3A, Table 3). DCA showed that the nomogram had a greater net clinical benefit than that of the other scoring systems within a wide range of threshold probabilities (Fig. 3B).

**Table 3:** Predictive performances and validation of the nomogram

Model	C index (95%CI)	*P* value	NRI (95%CI)	*P* value	IDI (95%CI)	*P* value
ESM-based nomogram	0.836(0.798–0.873)	< 0.001				
Base model	0.803(0.761–0.845)	< 0.001	0.132(0.044–0.217)	0.003	0.051(0.030–0.071)	< 0.001
CURB-65	0.669(0.622–0.715)	< 0.001	0.882(0.715–1.048)	< 0.001	0.237(0.197–0.277)	< 0.001
PSI	0.704(0.654–0.754)	< 0.001	0.558(0.380–0.735)	< 0.001	0.192(0.138–0.246)	< 0.001
APACHEII	0.740(0.693–0.786)	< 0.001	0.426(0.246–0.606)	< 0.001	0.141(0.083–0.198)	< 0.001
SOFA	0.778(0.737–0.819)	< 0.001	0.296(0.114–0.479)	< 0.001	0.120(0.060–0.181)	< 0.001

*Abbreviations: NRI* Net reclassification improvement, *IDI* Integrated discrimination improvement, *CI* Confidence interval, *PSI* Pneumonia Severity Index, *APACHEII* Acute Physiology and Chronic Health Evaluation II, *SOFA* Sequential Organ Failure Assessment

## Discussion

In this study, we constructed a new nomogram based on ESM to predict in-hospital mortality among older patients with SCAP. This nomogram, which incorporated ESM into the base model and included routine clinical characteristics and laboratory indicators, showed a significantly better predictive performance than that of CURB-65, PSI, APACHE II, and SOFA. To our knowledge, this is the first report of a multivariable predictive model that includes skeletal muscle mass measurements and other readily available parameters in older patients with SCAP.

SCAP is associated with significant morbidity and mortality and poses a major threat to older adults. To date, several scoring systems and models have been developed to predict adverse outcomes of SCAP; however, none have been fully validated or ready for implementation in clinical practice. Among these models and scoring systems, CURB-65 and PSI are the most widely used prognostic prediction systems. However, their performance for in-hospital mortality (AUCs < 0.7) is not optimal in older adult patients with SCAP [17, 21, 22]. Several studies have shown that the CURB-65 has limited negative and positive predictive values, especially in patients aged 70 years and over [23, 24]. Consistent with previous studies, our study revealed low predictive abilities for both CURB-65 and PSI, which had AUCs of 0.669 (95% CI, 0.622– 0.715) and 0.704 (95% CI, 0.654–0.754), respectively. This result was attributed to our study population, which consisted of older patients with a median age of 80.0 years (IQR, 16.0). Although some studies have reported that ICU scoring systems, such as the APACHE II and SOFA, are superior to other severity scores in predicting SCAP mortality or CAP disease severity [25, 26], these systems are complicated and include some interventions that are not feasible within 24 h of SCAP onset.

The older population is growing rapidly worldwide [27], and it is essential to develop a specific assessment tool for older patients with SCAP. Previous studies have suggested that age-related parameters, including functional status, frailty assessment, malnutrition, and other geriatric syndromes, are strongly associated with poor prognosis in patients with CAP [28–30]. Sarcopenia is a relatively newly recognized geriatric syndrome in the geriatric population; therefore, minimal evidence is available regarding the association between sarcopenia and pneumonia [31]. One possible reason for this is that patients with SCAP are critically ill and unable to complete grip strength tests or the Short Physical Performance Battery. Although sarcopenia cannot be easily diagnosed in critically ill patients, muscle measurements can be readily obtained using CT scans. Additionally, bed rest and physical inactivity during hospitalization contribute to reduced muscle mass and decreased functional capacity in older adults. Low skeletal muscle mass has been associated with a poor prognosis of SCAP. Therefore, in our study, the measurement of muscle mass derived from chest CT was added to a multivariable model to explore its predictive effect on SCAP. As sarcopenia is highly associated with frailty and functional aspects, we also integrated the CFS (due to its feasibility) and the Barthel index to identify the frailty and functional status (basic activities of daily living), respectively [32, 33]. However, these two measures were not included in our multivariable model. Rather, we incorporated ESMcsa into the base model. Incorporating ESMsca into the base model, along with other risk factors, significantly improved its predictive accuracy. These findings indicate that clinicians should be mindful of muscle mass as determined using chest CT scans in clinical practice.

Our nomogram also included MAP, SpO_2_, GCS, LDH, lactate, and BUN. As components of the vital signs on admission, MAP and SpO_2_ data were readily available. Other studies have indicated that the systolic blood pressure or oxygenation index is associated with a poor prognosis of CAP [34, 35], which is consistent with our results. The GCS is the most widely used tool for consciousness assessment. Several studies have demonstrated that GCS is an independent prognostic factor for hospital mortality in ICU patients with lung infections [21, 35, 36]. LDH is a cytoplasmic enzyme that is widely distributed in major organs, including the lungs, kidneys, and skeletal muscles, and elevated serum LDH levels have been associated with SCAP and other respiratory diseases [37, 38]. The lactate level at admission is considered a rapid, inexpensive, and broadly available prognostic predictor that is independent of the CURB-65 scores for patients with CAP [22]. High lactate levels indicate organ failure and hypoperfusion and have been suggested as a guide to fluid resuscitation in critically ill patients [39, 40]. Furthermore, septic shock is defined as hyperlactatemia (lactate level > 2 mmol/L), and requires vasopressor administration to maintain a MAP of 65 mmHg [41]. Consistent with previous models and pneumonia severity scores such as the CURB-65 and PSI, our study showed that BUN was an important predictor of SCAP. Owing to study population heterogeneity, different predictive models have included various indicators of mortality in patients with SCAP.

Several limitations should be considered when interpreting the results of our study. First, this study was carried out in a single center with a small cohort of patients and chest CT is performed in most patients with pneumonia but not all, potentially contributing to selection bias. Second, not all potential risk factors were analyzed in our study, and some intervention parameters pertaining to mechanical ventilation and the use of vasopressors and antibiotics were excluded; these variables may not have been obtained in the early hospitalization course. Last, although the predictive accuracy of the nomogram was proven through internal validation, the data lacked external validity. Multicenter data should be considered in future studies to improve the robustness and performance of the nomogram model.

## Conclusion

A novel nomogram, which included MAP, SpO_2_, GCS, LDH, lactate, BUN, and ESMcsa determined from chest CT, was developed and validated to predict in-hospital mortality among older patients with SCAP. The predictive nomogram had a superior performance compared to the CURB-65, PSI, APACHE II, and SOFA scores. Our nomogram is a potentially useful tool that may help clinicians perform medical interventions in a timely manner and prevent adverse outcomes in older patients with SCAP.

### Supplementary Information

**Additional file 1.** TRIPOD Checklist: Prediction Model Development.

## Data Availability

The data used during the current study are available from the corresponding author upon reasonable request.

## Abbreviations

APACHE II: Acute Physiology and Chronic Health Evaluation II
BUN: Blood urea nitrogen
CAP: Community-acquired pneumonia
C-index: Concordance index
CFS: Clinical Frailty Scale
ESMcsa: Cross-sectional area of erector spinae muscle
ESM: Erector spinae muscle
ICU: Intensive care unit
IDI: Integrated discrimination improvement
GCS: Glasgow coma scale
LDH: Lactate dehydrogenase
MAP: Mean arterial pressure
NRI: Net reclassification improvement
PSI: Pneumonia Severity Index
SCAP: Severe community-acquired pneumonia
SOFA: Sequential Organ Failure Assessment
SpO_2_: Peripheral capillary oxygen saturation
